# Effects of Tai Chi Exercise on Reducing Falls and Improving Balance Performance in Parkinson's Disease: A Meta-Analysis

**DOI:** 10.1155/2019/9626934

**Published:** 2019-02-21

**Authors:** Hsin-Hsuan Liu, Nai-Chen Yeh, Yi-Fan Wu, Yea-Ru Yang, Ray-Yau Wang, Fang-Yu Cheng

**Affiliations:** ^1^Department of Physical Therapy and Assistive Technology, National Yang-Ming University, Taipei City 112, Taiwan; ^2^Institute of Long-Term Care, Mackay Medical College, New Taipei City 252, Taiwan

## Abstract

**Introduction:**

Parkinson's disease (PD) is a common neurodegenerative disorder that may increase the risk of falls, functional limitation, and balance deficits. Tai Chi was used as an option for improving balance in people with PD. The aim of this meta-analysis was to evaluate the effects of Tai Chi on falls, balance, and functional mobility in individuals with PD.

**Method:**

The literature search was conducted in PubMed, the Cochrane Library, CINAHL, PEDro, Medline, Embase, sportDISCUS, Trip, and the National Digital Library of Theses and Dissertations in Taiwan. Randomized controlled trials (RCTs) analyzing the effects of Tai Chi, compared to no intervention or to other physical training, on falls, functional mobility, and balance in PD patients were selected. The outcome measurements included fall rates, Berg Balance Scale (BBS), Functional Reach (FR) test, and the Timed Up and Go (TUG) test. Two reviewers independently assessed the methodological quality and extracted data from the studies using the PEDro scale.

**Results:**

Five RCTs that included a total of 355 PD patients were included in this review. The quality of evidence in these studies was rated as moderate to high. Compared to no intervention or other physical training, Tai Chi significantly decreased fall rates (odds ratio = 0.47, 95% confidence interval (CI) 0.30 to 0.74, and *p*=0.001) and significantly improved balance and functional mobility (BBS mean difference (MD) = 3.47, 95% CI 2.11 to 4.80, and *p* < 0.001; FR MD = 3.55 cm, 95% CI 1.88 to 5.23, and *p* < 0.001; TUG MD = −1.06 s, 95% CI −1.61 to −0.51, and *p* < 0.001) in people with PD.

**Conclusion:**

This meta-analysis provides moderate- to high-quality evidence from five RCTs that Tai Chi could be a good physical training strategy for preventing falls and improving balance and functional mobility in people with PD.

## 1. Introduction

Parkinson's disease (PD) is a common neurodegenerative disorder of the central nervous system that mainly affects the motor system. There are four typical motor symptoms of PD: rigidity, tremor/shaking, bradykinesia/akinesia, and postural instability/balance dysfunction that result from the death of neurons in the substantia nigra. In addition, nonmotor symptoms that include cognitive impairments and emotional and behavior problems may also occur in individuals with PD [1, 2]. According to previous studies, balance dysfunction, poor functional mobility, and cognitive impairments are primary causes that may increase the incidence of falls in people with PD [3–6]. Falls are common events in PD patients and are one of the major concerns related to their health status and quality of life. Approximately 45% to 68% people with PD fall every year, and two-thirds of them experience recurrent falling [7]. Moreover, falls can cause injuries, loss of functional independence in daily living, and even death [8].

There is growing evidence showing that exercise and physiotherapy can improve the performance of balance-related activities in patients with PD [9]. In this review, it was found that treadmill training, progressive resistance exercise, and aquatic therapy positively affected the balance ability of PD patients. Additionally, in this review, Tai Chi showed higher quality evidence that the performance of the Timed Up and Go (TUG) test, the Berg Balance Scale (BBS), and Functional Reach test can be improved after 12 to 24 weeks of Tai Chi training [9–12]. Tai Chi is a traditional calisthenics exercise practiced for defense and health benefits in the Chinese society. Tai Chi is also a balance-based exercise that links many slow and rhythmic movements together in a continuous sequence, and the center of gravity (COG) moves with the movements of each foot. The practice of this exercise involves lower limb control, lower limb strengthening, and dynamic posture control. People who practice Tai Chi maintain different postures and keep the COG within a changing base of support to challenge their balance control system [13]. A previous study indicated that an increased risk of falls is related to postural instability in individuals with PD. Since Tai Chi can enhance the dynamic postural stability, Tai Chi can be suggested as an effective way to reduce falls in individuals with PD [14, 15].

Previous systematic reviews and meta-analyses indicated many benefits of Tai Chi on balance, motor function, and gait performance in people with PD. However, most of the outcomes of these review studies were used to evaluate balance-related activities or fall-related predictors, such as the BBS, the TUG, and the Sit-to-Stand tests. However, these outcomes cannot exactly reflect the real situation of falls. To our knowledge, there are no meta-analyses identifying the efficacy of Tai Chi on reducing the fall rate in individuals with PD. Therefore, this meta-analysis aimed at investigating the actual effects of Tai Chi training on falls and at evaluating the effects of Tai Chi on functional mobility and balance in people with Parkinson's disease.

## 2. Materials and Methods

### 2.1. Study Design and Registration

This meta-analysis adhered to the Preferred Reporting Items for Systematic Reviews and Meta-Analyses (PRISMA) statement and is registered with PROSPERO (registration number: CRD42018073565).

### 2.2. Literature Search and Study Selection

We searched PudMed, the Cochrane Library, Medline, Embase, PEDro, CINAHL, SportDISCUS (EBSCO), Airiti Library, and Trip from inception through May 31, 2018, using a combination of the following MeSH search terms: [(Parkinson “AND” Tai Chi “OR” Tai Ji “AND” balance “OR” fall)]. The reference lists of the included studies or relevant reviews were screened manually for additional studies. Three authors of this study independently screened the titles and abstracts of all studies identified through the database searches. Eligible studies were included for study if they were randomized controlled trials (RCTs) that were published in English or Chinese. Studies were included in this meta-analysis if they included participants with PD.

We included clinical trial studies in which PD patients participated in Tai Chi exercises and in which the participants were not given any particular instructions about performing Tai Chi outside of class. We compared PD patients who had participated in Tai Chi to control PD patients who had received regular exercises (RE), physical treatments (i.e., resistance training, balance training, and strengthening), or no treatment. The primary outcome measure for this meta-analysis was the number of participants with PD who experienced a fall. The secondary outcomes included the Berg Balance Scale (BBS), the Forward Functional Reach (FR) test, and the Timed Up and Go (TUG) test.

### 2.3. Study Quality

The Physiotherapy Evidence Database (PEDro) scale was used in this study to assess the methodological quality of all the included studies. Each article was evaluated by at least two reviewers (HHL and NCY). A third independent reviewer (YFW) would be consulted to resolve disagreements if there was any argument. The PEDro scale is a valid measure of the methodological quality of clinical trials in the physical therapy field [16]. The PEDro scale contains 10 items (specified eligibility criteria): random allocation, concealed allocation, baseline similarity, blinding of subjects, blinding of therapists, blinding of assessors, measures of key outcomes from more than 85% of subjects, intention-to-treat analysis, between-group statistical comparisons, and point measures and measures of variability. Items are scored as either “yes” (1) or “no” (0), and a score out of 10 is obtained by summation. The area study is considered to be high quality if the score is above 6. Scores of 4 and 5 are interpreted as moderate quality, and scores less than 4 are interpreted as poor quality.

### 2.4. Statistical Analysis

Each outcome was analyzed by using the Review Manager statistical software (RevMan 5.3 version, Cochrane, USA). We evaluated the heterogeneity among studies with the *I*^2^-index statistic. A value of *I*^2^ > 50% accompanied by *p* < 0.1 for the heterogeneity test was indicated as a moderate to high level of heterogeneity, and therefore, a random effects model was used. In contrast, a fixed effects model was used in instances of minimal heterogeneity (*I*^2^ < 50%, *p* > 0.1).

## 3. Results

### 3.1. Search Results

**Figure 1:**
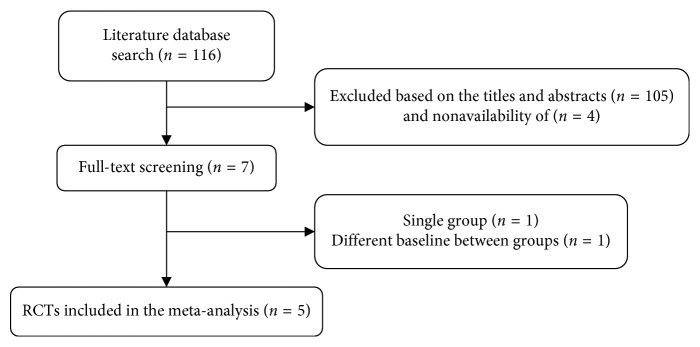
Flow diagram of study selection and identification. This meta-analysis included 5 randomized controlled trials and excluded based on the titles and abstracts (*n*=105).

The electronic search and hand search for additional resources identified 116 potential records. After eliminating duplicates and excluding studies based on the titles and abstracts, the number of relevant records was reduced to 11. Among the 11 potentially eligible studies, 4 studies were excluded due to missing full text. After reviewing the remaining 7 full-text articles, 2 were rejected, one article was rejected for reporting on a single group design study, and the other because the groups were not similar at baseline. Finally, five studies were included in the meta-analysis (Figure 1).

### 3.2. Study Characteristics and Quality

**Figure 2:**
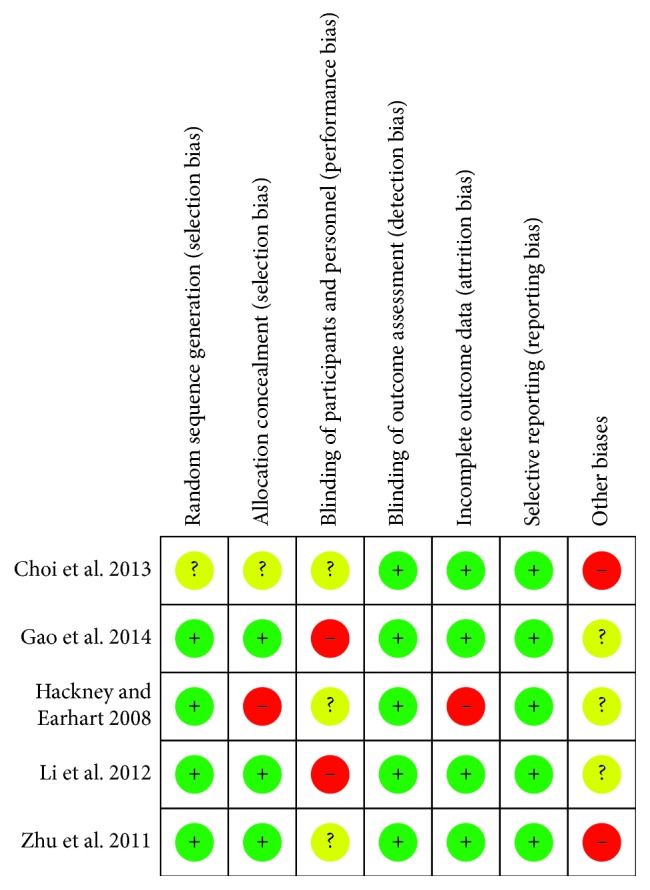
Risk of bias of the included studies (*n*=5).

**Figure 3:**
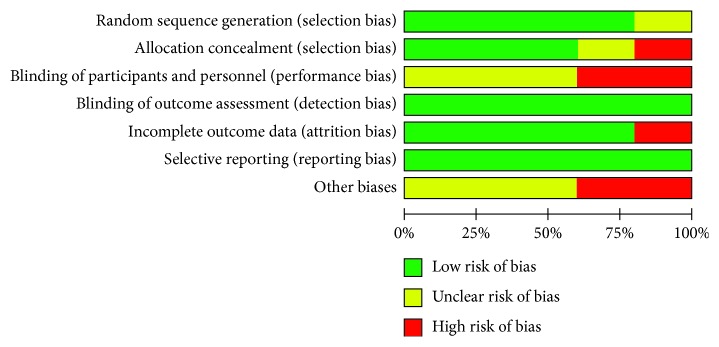
Summary of the risk of bias. The overall risk of bias, except for blinding (performance bias), was low.

The characteristics of the 5 included studies are summarized in Table 1. The studies were published in China, Korea, and the United States between 2008 and 2014. The sample size of these studies ranged from 20 to 195 (total number of participants = 355). The mean age range of the included participants with PD was between 40 and 85 years, and the disease severity was between 1 and 4 in terms of the Hoehn and Yahr staging system. The interventions in the experimental groups were Tai Chi exercises, while the comparisons included no intervention, stretching/resistance training, and walking. Most of the treatment schedules in the included studies were set at 60 min for each session, 2-3 times per week, and the total treatment duration ranged from 4 weeks to 24 weeks. The outcome measures included the number of participants with PD who experienced a fall, the Berg Balance Scale (BBS), the Forward Functional Reach (FR) test, and the Timed Up and Go (TUG). The risk of bias is summarized in Figures 2 and 3. The study quality, as assessed by the PEDro scale [17], is shown in Table 2 (average score = 6.4, range = 5–8).

### 3.3. Effects of the Interventions

**Figure 4:**
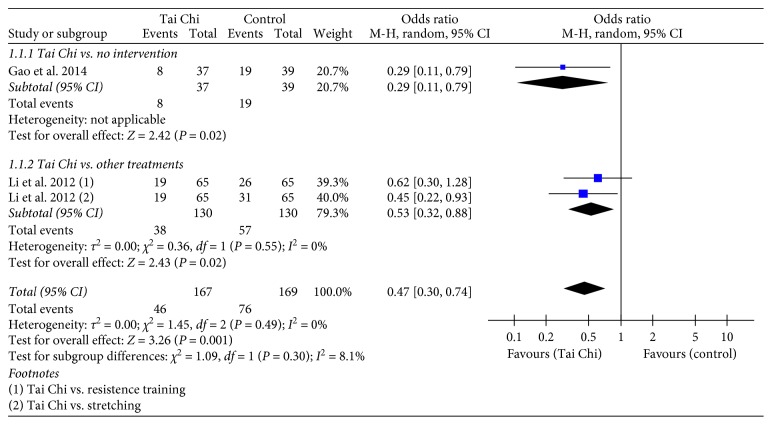
Forest plot showing the effect of Tai Chi on the number of participants with PD who experienced a fall.

There were two studies (*n*=275) that reported the number of participants with PD who experienced a fall and the number of participants with PD who did not experience a fall [12, 18]. Compared to the groups that received no intervention, the Tai Chi groups had a significantly reduced number of participants with PD who experienced a fall (*n*=76, OR = 0.29, 95% CI = 0.11–0.79, and *p* < 0.05) [12]. The Tai Chi groups also showed greater reductions in the number of participants with PD who experienced a fall than the other treatments, such as resistance training and stretching (*n*=195, OR = 0.53, 95% CI = 0.32–0.88, and *p* < 0.05) [18] (Figure 4).

**Figure 5:**
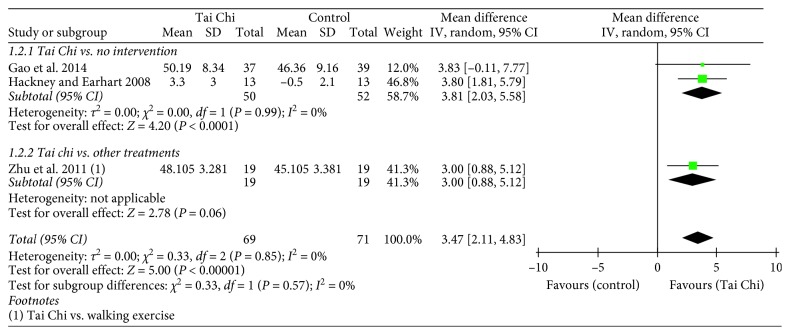
Forest plot showing the effect of Tai Chi on the Berg Balance Scale (BBS) in individuals with PD.

A total of three studies (*n*=140) assessed balance function by using BBS [10, 12, 19]. The balance function was significantly improved in the Tai Chi groups compared to that in the no intervention group (*n*=102, MD = 3.81, 95% CI = 2.03–5.58, and *p* < 0.0001) [10, 12] and the walking exercise group (*n*=38, MD = 3.00, 95% CI = 0.88–5.12, and *p* < 0.05) [19] (Figure 5).

**Figure 6:**
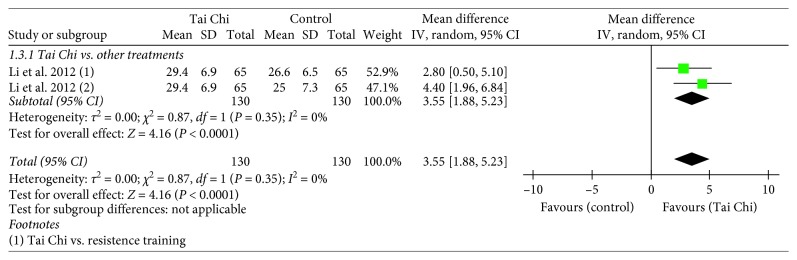
Forest plot showing the effect of Tai Chi on the Forward Functional Reach (FR) test in individuals with PD.

Only one study examined the effect of the Tai Chi intervention on the Functional Reach (FR) test, and the study reported a significantly better performance in the Tai Chi group compared to that of the other intervention groups, such as resistance training and stretching (*n*=195, MD = 3.55, 95% CI = 1.88–5.23, and *p* < 0.0001) [18]. (Figure 6).

**Figure 7:**
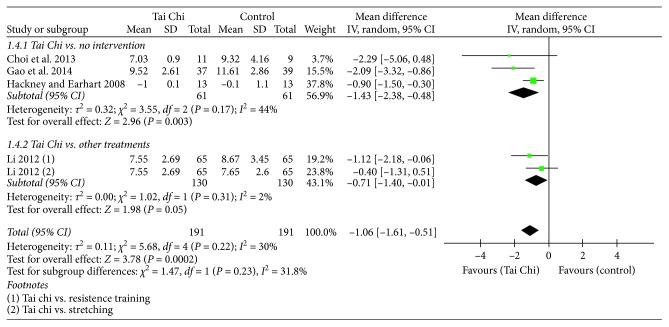
Forest plot showing the effect of Tai Chi on the Timed Up and Go (TUG) in individuals with PD.

Four of the five studies (*n*=317) assessed functional mobility using the TUG [10, 12, 18, 20]. The Tai Chi groups demonstrated a significantly reduced completion time of the TUG compared to that of the no intervention group (*n*=122 MD = −1.43, 95% CI = −2.38–(−0.48), and *p* < 0.05). The Tai Chi group demonstrated a trend of better functional mobility compared to other treatments such as resistant training and stretching, but it was not significant (*n*=195, MD = −0.71, 95% CI = −1.40–(−0.01), and *p* < 0.05) (Figure 7).

## 4. Discussion

The aim of this meta-analysis was to evaluate the effects of Tai Chi on falls, functional mobility, and balance in people with PD. The present meta-analysis found that Tai Chi, compared to no intervention or other exercises, can significantly reduce the number of participants with PD who experienced a fall. Significant improvements in functional mobility and balance were also observed in the Tai Chi groups compared to the control groups that received no intervention or active therapies. These findings were obtained from a pooled analysis of five RCTs that included a total of 355 patients.

The meta-analysis result from this study reported that the ratio of the number of participants with PD who experienced a fall to the number of participants who did not experience a fall was significantly lower in the Tai Chi group than in the control group (Tai Chi/odcontrol = 46 : 121/76 : 93, ds ratio = 0.47). A previous study showed that technology-assisted balance and gait training could also decrease falls in patients with PD, and the odds ratio was 0.109 (technology-assisted/control = 2 : 20/11 : 12) [21]. From the results of this analysis, we were further convinced of the effect of Tai Chi on reduced incidences of falling in individuals with PD.

Tai Chi exercises were beneficial for the balance and functional mobility of people with PD, as detected by the BBS and FR tests. This result was consistent with the findings of a prior review that aimed at evaluating the effects of Tai Chi on balance function in an elderly population [22]. According to Li et al., a significant prepost change in FR was observed after participating in Tai Chi exercises [23]. This result suggested that Tai Chi can be provided as an appropriate approach for older adults with PD to improve and maintain balance function.

According to the meta-analysis result of the present study, the improvement in the TUG in the Tai Chi group was significantly better than that in the no intervention group but was not better than that in the resistance training group. TUG involves several movement components such as standing up from a chair, walking three meters, turning around, walking back to the chair, and sitting down. People not only need good balance function but also sufficient muscle strength and power in their lower extremities to execute this task [24, 25]. Therefore, the nonsignificant better effect of Tai Chi was observed when the control group received resistance training.

Tai Chi is associated with great benefits for the balance and functional mobility of patients with PD. These benefits can further reduce the fall rate in the PD population, which is supported by the present meta-analysis results. These beneficial effects may be related to certain characteristics of Tai Chi exercise. First, Tai Chi includes many movements with slow weight shifting, body rotation, and single-leg standing in different positions, requiring delicate joint control with muscle coordination [26]. In patients with PD, the loss of anticipatory postural control may affect their balance function [19]. Tai Chi emphasizes slow speed movements, which may provide more time for motor programming and execution. Second, Tai Chi exercise may provide more proprioceptive stimulation and lower extremity muscle strengthening, which are important for movement control in daily life. Additionally, flexibility and coordination of the whole body can be improved after Tai Chi exercise [27, 28]. Finally, recent studies also found that Tai Chi practitioners showed better cognitive functions, such as motor learning, memory, and executive function [29]. Overall, the positive effects on balance, motor function, coordination, muscle strength, flexibility, and cognitive function after Tai Chi exercise may prevent PD patients from falling.

Tai Chi was considered to be an alternative treatment to improve balance function in individuals with PD. However, the treatment protocol has not yet been described. In most of the included studies, the treatment duration and frequency were set at 60 min for each session, 2 to 3 sessions per week, for total 10 to 24 weeks. A previous review noted that 40 to 60 min of exercise, 2 to 3 days per week, for 12 weeks, may be an effective dose to benefit postural stability in the PD population [30]. However, a longer duration of Tai Chi exercise may increase and expand the benefit [10].

It is worth mentioning that all the studies included in this meta-analysis only included American and Asian participants. Therefore, the effects in these studies cannot be generalized to populations from other areas.

There are some limitations to our study. First, a small number of eligible studies were included in the meta-analysis in the present review. Four studies were excluded due to unavailable full text. Second, the latest study included in our meta-analysis was published in 2014. Third, we only included articles that were published in Chinese or English, which may have resulted in a language bias. Finally, there were no studies examining the effects of Tai Chi on PD patients with different Hoehn and Yahr stages.

## 5. Conclusion

This meta-analysis provides moderate- to high-quality evidence from five RCTs that Tai Chi could be a good physical training strategy for preventing falls and improving balance and functional mobility in people with PD. Further studies should investigate the effects of Tai Chi and the precise intervention protocols for PD patients with different disease stages.

## Figures and Tables

**Table 1:** Characteristics of the included studies.

Study	Participants	Interventions	Outcomes
Choi et al. 2013 [20]	*n*=20 Hoehn and Yahr scale 1-2 stage Stable drug regimen	Tai Chi vs. no intervention 60 min, 3 times/week, 12 weeks	*Timed Up and Go (TUG)* UPDRS III, OLS, 6 MWT, physical function (lateral stance, agility, tandem gait)
Gao et al. 2014 [12]	*n*=76 Age > 40 y/o Independent walking ≥ 1 fall during past 1 y	Yang style Tai Chi vs. no intervention 60 min, 3 times/week, 12 weeks	*Occurrences of falls, Berg Balance Scale (BBS), TUG*, UPDRS III
Hackney and Earhart 2008 [10]	*n*=26 Age > 40 y/o Hoehn and Yahr scale 1.5–3 stage independent walking with/without aids for 3 min	Yang short style Tai Chi vs. no intervention 60 min, 2 times/week, 20 sessions	*BBS, TUG* UPDRS III, tandem stance (TS), one-leg stance (OLS), GAITRite, 6 MWT
Li et al. 2012 [18]	*n*=195 Age: 40–65 y/o Hoehn and Yahr scale 1–4 stage ability to walk with/without aids	Tai Chi vs. resistance training v.s. stretching 60 min, 2 times/week, 24 weeks	*Falls, TUG, Functional Reach (FR) test*, UPDRS motor scores limit-of stability test, gait, strength
Zhu et al. 2011 [19]	*n*=38 Age: 40–85 y/o Hoehn and Yahr scale 1-2 stage onset < 3 y	Tai Chi vs. walking exercise 30–45 min, 2 times/day, 5 days/week, 4 weeks	*BBS* UPDRS III

**Table 2:** Quality of the included studies assessed by the PEDro scale. The highest score was 8 out of 10. The average score in this meta-analysis was 6.4, which is considered moderate- to high-quality evidence.

Study	Random allocation	Concealed allocation	Groups similar at baseline	Participant blinding	Therapist blinding	Assessor blinding	<15% dropouts	Intention-to-treat analysis	Between-group difference reported	Point estimate and variability reported	Total
Choi et al. 2013 [20]	Y	N	Y	N	N	Y	N	N	Y	Y	5
Gao et al. 2014 [12]	Y	Y	Y	N	N	Y	Y	N	Y	Y	7
Hackey and Earhart 2008 [10]	Y	N	Y	N	N	Y	N	N	Y	Y	5
Li et al. 2012 [18]	Y	Y	Y	N	N	Y	Y	Y	Y	Y	8
Zhu et al. 2011 [19]	Y	Y	Y	N	N	Y	Y	N	Y	Y	7
